# Application of Artificial Intelligence in the Interpretation of Pulmonary Function Tests

**DOI:** 10.7759/cureus.82056

**Published:** 2025-04-11

**Authors:** Talha Saad, Ramesh Pandey, Surendra Padarya, Puja Singh, Satish Singh, Nitu Mishra

**Affiliations:** 1 Department of Pulmonary Medicine, Bundelkhand Medical College, Sagar, IND; 2 Department of Medicine, Bundelkhand Medical College, Sagar, IND; 3 Department of Orthopedics, Bundhelkhand Medical College, Sagar, IND; 4 Department of Pathology, Jawaharlal Nehru Medical College, Wardha, IND; 5 Department of Pathology, Bundelkhand Medical College, Sagar, IND; 6 Department of Information Technology, Corot Systems Private Limited, Noida, IND; 7 Department of Microbiology, Indian Council of Medical Research, Sagar, IND

**Keywords:** artificial intelligence, chronic obstructive pulmonary disease, pft, pulmonary function test, spirometry

## Abstract

Background

As per the Global Burden of Disease Study (GBD) 2019, chronic obstructive pulmonary disease (COPD) and asthma had a significant global burden. COPD is the fourth leading cause of death in the world and the second leading cause of death and disability-adjusted life years (DALYs) in India. Pulmonary function tests (PFTs) are commonly used diagnostic tools. They include spirometry, body plethysmography, and diffusion capacity. In regions with limited resources, pulmonologists often only have access to spirometry. Additionally, PFT pattern interpretation is usually unreliable and subjective. Recent rapid advances in artificial intelligence (AI) algorithms can bridge the gaps.

Objectives

This study aims to compare the accuracy of the predictions made by AI algorithms with pulmonologists using limited clinical data and spirometry. It also examines the consistency and accuracy of pulmonologists' predictions based on the same information.

Methodology

Different AI algorithms were trained, and their accuracy was evaluated. Spirometry and limited clinical data from 440 patients were interpreted by an AI algorithm and eight senior pulmonologists. Accuracy, sensitivity, specificity, positive predictive value (PPV), and negative predictive value (NPV) were calculated for the different patterns.

Results

Approximately 60% of the cases involved male patients, and about 70% were between the ages of 21 and 60. The Fleiss's kappa was 0.46. While the accuracy of pulmonologists against the gold standard was 65.82%, the accuracy of the AI was 86.59%.

Conclusions

PFTs, when interpreted by pulmonologists with limited clinical and spirometry data, have lower accuracy and higher variability. AI algorithms can consistently produce high accuracy. Adopting such technology among clinicians, especially in resource-constrained regions, could be pivotal for offering quality healthcare. In addition, it will also help in getting rid of inter-observer variability.

## Introduction

Chronic obstructive pulmonary disease (COPD) is a progressive respiratory condition with major sequelae, including cardiovascular risk, respiratory failure, and lung cancer. COPD inflicts a large and growing burden in both direct and indirect costs to society [1]. According to the World Health Organization (WHO), COPD is the fourth leading cause of death in the world, a slip of one place due to COVID-19. It causes 5% of the total deaths reported worldwide [2]. According to the Global Burden of Disease (GBD) report, COPD is the second leading cause of death and disability-adjusted life years (DALYs) in India [3].

According to the GBD Study 2019, 262 million people are affected by asthma, i.e., 3,416 cases per 100,000. Also, approximately 461,000 people worldwide died from asthma [4].

Due to the global impact of asthma and COPD, a quick and reliable diagnostic tool for pattern interpretation of PFT results is needed. Traditionally, the Pulmonary Function Test (PFT) is widely used for diagnosing asthma and COPD. Pulmonologists use the Global Initiative for Chronic Obstructive Lung Disease (GOLD) guidelines to interpret the PFT results. PFT includes spirometry, body plethysmography, and diffusion capacity. In regions with limited resources, often, only spirometry is accessible for pulmonologists to work with. In addition, PFT results depend on breathing patterns; hence, they are often unreliable and subjective [5]. Rapid advances in artificial intelligence (AI) algorithms can fill the shoes.

This study aims to explore the possibility of leveraging the accuracy of the machine learning/AI algorithm to predict the pulmonary function test pattern using limited clinical data and spirometry. It also explored the consistency and accuracy of predictions made by pulmonologists.

## Materials and methods

Study design

This retrospective-prospective, observational, and analytical study was conducted to assess the viability of using AI to interpret pulmonary function tests. A total of 3,230 retrospective cases (from January 1, 2012, to February 28, 2023) and 440 prospective cases (from March 15, 2023, to August 15, 2024), reported at Bundelkhand Medical College, Sagar Hospital, were included in this study. All the retrospective cases were used to train the AI algorithms, and prospective cases were used to evaluate the performance of the AI algorithms against pulmonologists.

Ethical clearance

The study was conducted after receiving approval from the Institutional Ethics Committee, Bundelkhand Medical College, Sagar. 

Sample size

This study collected prospective data, including complete pulmonary function tests (PFTs), clinical information, X-rays, and other additional investigations, from 440 patients over 17 months at a tertiary care center in central India. Eight senior pulmonologists then analyzed the data as per the study protocol, resulting in 3,520 independent data points (440 × 8). In addition, 3,230 retrospective cases were used for training AI algorithms.

Inclusion criteria

All patients over 18 years of age who underwent pulmonary function tests, regardless of gender, and for whom the following information was available were included in the study: comprehensively and accurately recorded PFT results, well-documented clinical details, and available X-ray and other clinical test records.

Exclusion criteria

The following types of cases were excluded from the study: patients with diagnosed respiratory conditions who did not undergo pulmonary function tests; patients whose PFT results were incomplete or lacked detail for any reason; patients who did not provide written consent for participation in the research; and cases involving other conditions such as lung cancer, cardiovascular diseases, or ear, nose, and throat disorders.

Consent requirement

Written consent was obtained from all prospective study participants. In all cases, the information was depersonalized before being shared among the pulmonologists via Google Forms.

Classification guidelines

This study classified the PFT spirometry patterns into normal, obstructive, and others. The other category included both restrictive and mixed cases. The severity of the obstructive cases was evaluated as mild, moderate, moderately severe, severe, and very severe. Since the severity of restrictive cases cannot be determined based solely on spirometry and requires additional parameters such as total lung capacity (TLC), the severity of these cases could not be assessed in this study. Similarly, to establish the mixed pattern, TLC is required and thus cannot be established in this study. The criteria for these classifications are covered in Table 1.

**Table 1:** Criteria to determine PFT pattern and severity based on spirometry. Source: [6]. FEV1, forced expiratory volume in one second; FVC, forced vital capacity; TLC, total lung capacity

PFT pattern	Severity	FEV1 of predicted value	FVC	FEV1/FVC	TLC
Obstruction	Mild	>70%	<80%	<70%	-
	Moderate	60%-69%	-	-	-
	Moderately severe	50%-59%	-	-	-
	Severe	35%-49%	-	-	-
	Very severe	<35%	-	-	-
Restriction		-	<80 %	>70 %	<80%
Normal		>80%	>80%	>70%	-
Mixed		-	<80 %	<70%	<80%

Gold standard diagnosis

To compare the interpretation of PFTs by two approaches, trained pulmonologists and AI, a benchmark needed to be established. For this purpose, data from patients who met the inclusion criteria were analyzed by an expert committee of two trained pulmonologists using complete PFT results, clinical history, and additional diagnostic tests. They classified each case based on PFT pattern interpretation into one of the following categories: normal, obstructive, restrictive, or mixed. They also assigned the severity of the obstructive pattern as mild, moderate, moderately severe, severe, and very severe. As part of this process, a total of 452 cases were reviewed by the committee, and 12 were excluded due to a lack of consensus on a common diagnosis.

AI-based algorithms development

Various AI algorithms, including Decision Trees, Support Vector Machines, K-Nearest Neighbors (KNN), Naïve Bayes, and Neural Networks, were developed and evaluated in this study. Retrospective data of PFTs for the last 10 years were used for these algorithms. The data were normalized to minimize bias and ensure faster convergence of the AI algorithms. It was then split into two sets: training and testing data, in an 80:20 ratio. The algorithms were trained on the training set, and their performance was evaluated using the test set. Fine-tuning was performed as needed to optimize performance. This iterative process was repeated till a maximum/satisfactory level of accuracy was achieved. The AI algorithms were developed using Python (v3.9) and its libraries, namely, Pandas (v1.3.5), Seaborn (v0.11.2), and SciPy (v1.7.1).

Comparative evaluation

Complete PFTs (pre-and/or post-bronchodilator spirometry) and limited clinical information (smoking history, cough, sputum, and dyspnea) were then independently evaluated by eight senior pulmonologists from six hospitals. Each week during the study period, data from 5 to 15 cases were shared with all pulmonologists via Google Forms (Appendix). They independently reviewed the cases and submitted their diagnoses for the PFT pattern interpretation and obstructive severity.

The data points provided to the pulmonologists were also fed into the AI algorithm to generate comparable diagnoses. The diagnoses made by both the pulmonologists and the AI algorithms were then assessed against the gold-standard diagnosis.

Data collection and processing

Reports of patients meeting the inclusion criteria were retrieved, digitalized, and depersonalized. Diagnoses made by both pulmonologists and AI algorithms were recorded in Microsoft Excel (Microsoft, Redmond, WA).

Spirometry tests

All spirometry tests were performed using standardized equipment (Pneumotrac Spirometer, Lenexa, KS) by respiratory technicians, by American Thoracic Society/European Respiratory Society (ATS/ERS) 2019 guidelines [7].

Statistical tests

To analyze central tendency, the mean with standard deviation and the median with range were calculated as appropriate. Since the number of raters (pulmonologists) was greater than two, inter-rater reliability (IRR) was assessed using Fleiss’s kappa. A box-and-whisker plot was used to represent the distribution of accuracy by rater. Confusion matrices were created for pulmonologists vs. gold standard and AI vs. gold standard. These matrices were used to evaluate accuracy and consistency, as well as to derive specificity, sensitivity, NPV, and PPV.

The statistical analysis was performed using Python (v3.9) and its libraries, including NumPy (v1.24), Pandas (v1.3.5), and Seaborn (v0.11.2).

## Results

Demographic and clinical distribution

The demographic distribution of the study cases is presented in Table 2. Among the *training data* used to train the AI algorithms, 1,943 (60.15%) cases were males. Approximately 2,616 cases (80.99%) were between the ages of 21 and 60, with the highest concentration in the 51-60 age group. According to BMI distribution, 3,041 cases (94.14%) had a BMI of less than 30. Among the study data, 257 (60%) were male. Around 300 (70%) were between the ages of 21 and 60, with the highest concentration in the 41-50 age group. According to BMI distribution, 417 (94.77%) of the cases had a BMI of less than 30. The average weight of patients was 46.25 ± 6.5 kg, and the average height was 166 ± 1.26 cm.

**Table 2:** Baseline demographic distribution of training (N = 3,230) and study data (N = 440). *P*-values were calculated using the chi-square test. BMI, body mass index

Characteristic	Training data ( *N* = 3,230)		Study data (*N* = 440)	
	*n* (%)	*P*-value	*n* (%)	*P*-value
Gender				
Male	1,943 (60.15%)	0	257 (58.41%)	4.19E-04
Female	1,287 (39.85%)		183 (41.59%)	
Age (years)				
00-20	340 (10.53%)	0	40 (9.09%)	5.73E-04
21-30	586 (18.14%)		72 (16.36%)	
31-40	572 (17.71%)		73 (16.59%)	
41-50	526 (16.28%)		81 (18.41%)	
51-60	592 (18.33%)		74 (16.82%)	
61-70	421 (13.03%)		51 (11.59%)	
71 or more	193 (5.98%)		49 (11.14%)	
BMI (kg/m²)				
<18.5	1,201 (37.18%)	0	138 (31.36%)	0
18.5-24.9	1,430 (44.27%)		201 (45.68%)	
25.0-29.9	410 (12.69%)		78 (17.73%)	
30-34.9	112 (3.47%)		18 (4.09%)	
35-39.9	62 (1.92%)		4 (0.91%)	
>40	15 (0.46%)		1 (0.23%)	

The distribution of the relevant clinical features (smoking, sputum, dyspnea, and cough) is presented in Table 3. Approximately 90%, i.e., 404, of the cases were currently smoking or had left smoking. Eighty-one percent of the cases were smoking for over five years. The highest number, 208 out of 440, of the smokers were smoking more than six cigarettes per day. The most common colors of sputum were dark yellow, red, and pink, with 153, 82, and 61 cases, respectively. Most often, the sputum was severely viscous, reported in 232 cases (52.73%). Blood was present in the sputum in 143 cases (32.5%), followed by purulent and mucous types, observed in 117 (26.59%) and 106 (24.09%) cases, respectively. Dyspnea was reported in 384 (87.27%) cases. The highest reported rating is 1 on the Modified Medical Research Council scale (mMRC) scale. About 264 (60%) of the cases reported Cough Severity Index (CSI) less than or equal to 3. 

**Table 3:** Distribution of cases for relevant clinical features (N = 440). Intensity of smoking is measured by the number of cigarettes per day. *P*-values calculated for distribution using the chi-square test. mMRC, Modified Medical Research Council scale; CSI, Cough Severity Index

Characteristic	Feature	Class	*n* (%)	*P*-value
Smoking	Current status	Never	36 (8.18%)	0
		Currently smoking	293 (66.59%)	
		Former	111 (25.23%)	
	Length of smoking	<2 years	24 (5.45%)	0
		2-5 years	58 (13.18%)	
		5-10 years	172 (39.09%)	
		>10 years	186 (42.27%)	
	Intensity of smoking	< 2	43 (9.77%)	0
		3-8	196 (44.55%)	
		> 8	201 (45.68%)	
Sputum	Color	Translucent	29 (6.59%)	0
		White	29 (6.59%)	
		Dark yellow	153 (34.77%)	
		Black	49 (11.14%)	
		Brown	37 (8.41%)	
		Pink	61 (13.86%)	
		Red	82 (18.64%)	
	Viscosity	Thin	67 (15.23%)	0
		Moderately viscous	141 (32.05%)	
		Severely viscous	232 (52.73%)	
	Consistency	Serous	74 (16.82%)	5.72E-05
		Purulent	117 (26.59%)	
		Mucous	106 (24.09%)	
		Blood	143 (32.5%)	
Dyspnea (mMRC scale)	0		56 (12.73%)	0
	1		152 (34.55%)	
	2		113 (25.68%)	
	3		51 (11.59%)	
	4		68 (15.45%)	
Cough (CSI)	≤3		264 (60%)	2.73E-05
	>3		176 (40%)	

The gold standard diagnosis of case distribution is presented in Table 4. Cases were categorized into four major patterns: normal, obstructive, restrictive, and mixed. The majority of cases fell under the obstructive pattern, accounting for 382 cases (86.82%). The most prominent severity of obstructive cases was mild, with 127 (33.25%) cases, followed by moderate, with 88 (23.04%) cases. 

**Table 4:** Distribution of cases based on gold standard diagnosis. The percentage for severity is based on total obstruction cases, i.e., 382.

Pattern	*n* (%)	Severity	*n* (%)
Normal	31 (7.04%)	-	-
Obstruction	382 (86.82%)	Mild	127 (33.25%)
		Moderate	88 (23.04%)
		Moderate severe	71 (18.59%)
		Severe	58 (15.18%)
		Very severe	38 (9.94%)
Restriction	15 (3.41%)	-	-
Mixed	12 (2.73%)	-	-

AI algorithm output analysis

**Figure 1:**
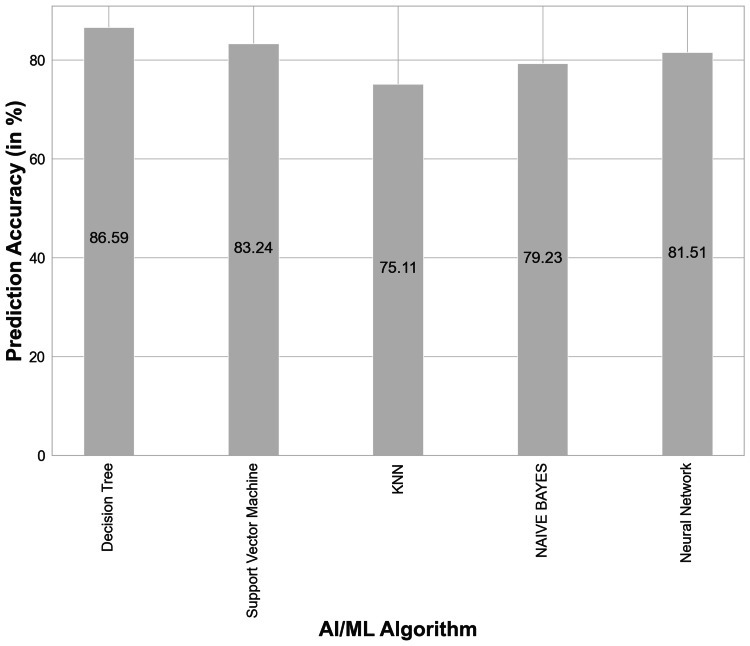
Prediction accuracy of different machine learning/artificial intelligence algorithms for PFT interpretation against gold standard (N = 440). Vertical lines on top of the bars represent errors. KNN, K-Nearest Neighbors; PFT, pulmonary function test; AL/ML, artificial learning/machine learning

Different AI algorithms for PFT interpretation were developed based on historical data from the last 10 years. These algorithms were then tested for their prediction accuracy against the gold standard on data from 440 cases collected for this study. Among these, the Decision Tree showed the best accuracy of 86.59%, followed closely by the support vector machine and perceptron with 83.24% and 81.51%, respectively. Prediction accuracy for different machine learning and AI algorithms is presented in Figure 1.

**Figure 2:**
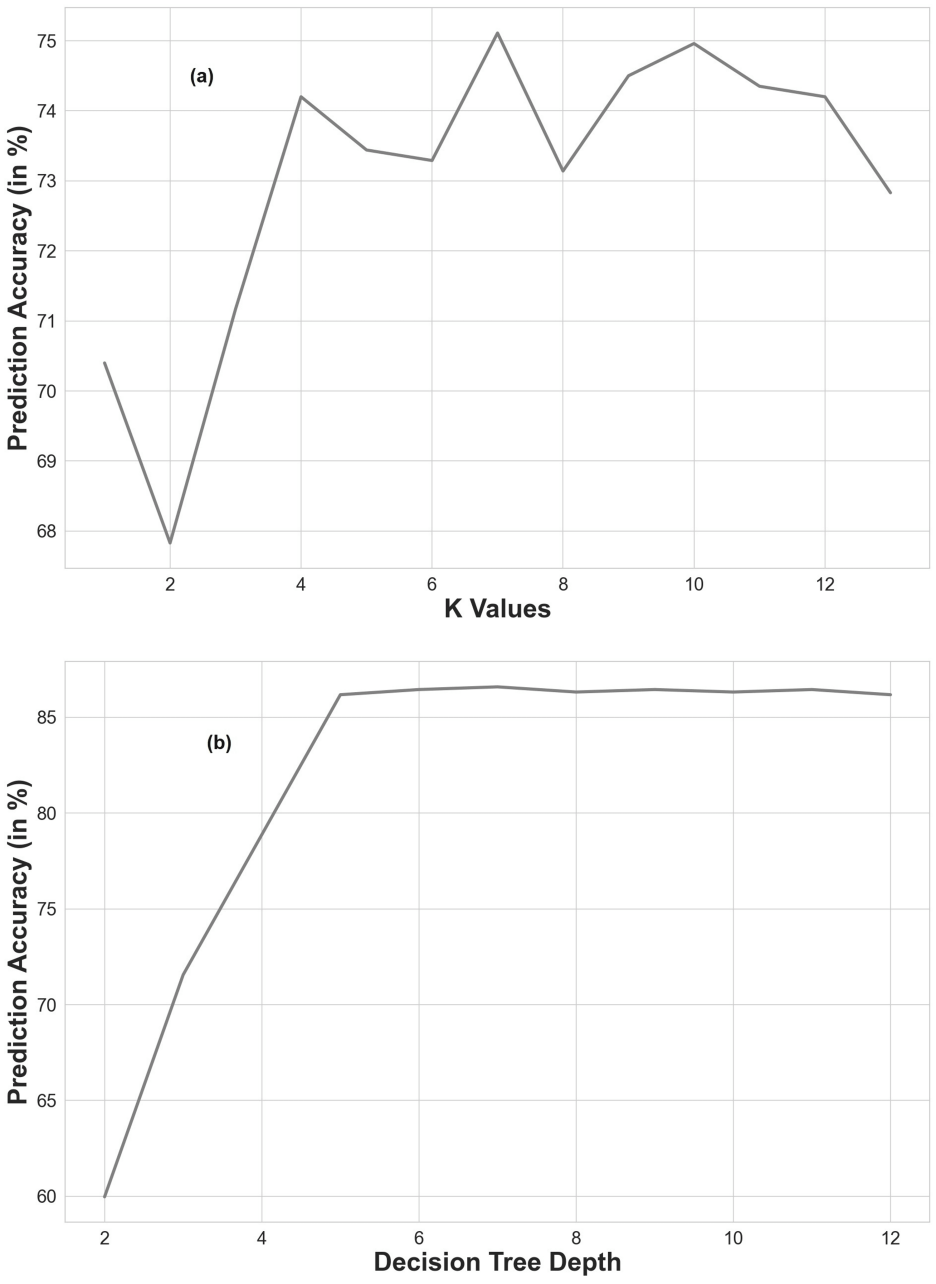
Variation in prediction accuracy with hyperparameter tuning of AI algorithms: (a) K-value for the KNN algorithm, (b) depth for the Decision Tree algorithm. KNN, K-Nearest Neighbors

Machine learning algorithms, KNNs and Decision Trees, were optimized to achieve maximum prediction accuracy. The KNN algorithm reached its highest accuracy at *K* = 7, while the Decision Tree performed best with a maximum depth of 4. The variation in prediction accuracy of these two algorithms upon fine-tuning is presented in Figure 2.

Inter-rater reliability

Fleiss's kappa was calculated to assess IRR among eight pulmonologists, as the variable was ordinal. The Fleiss's kappa (κ) was 0.46, which falls within the range of moderate agreement.

**Figure 3:**
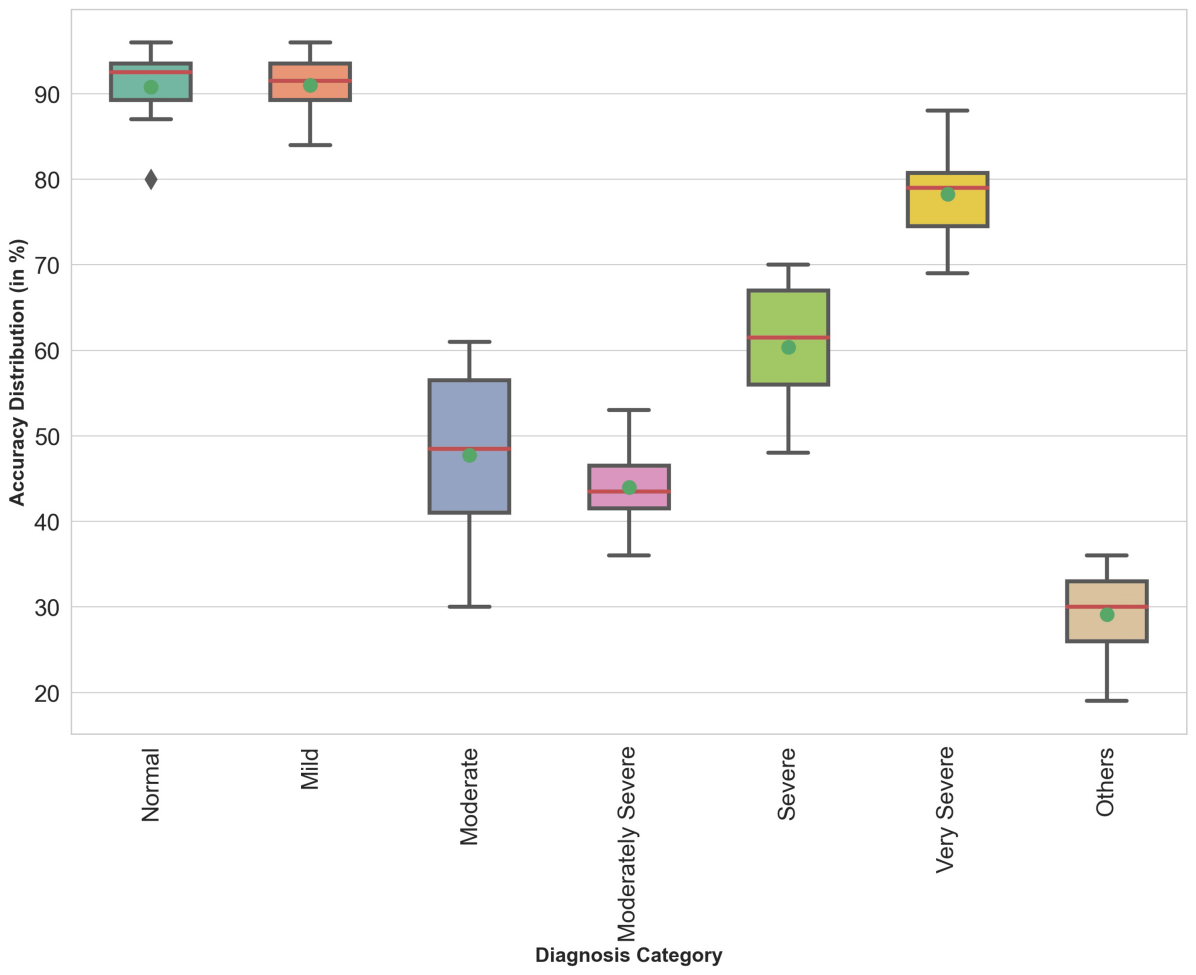
Variation in accuracy distribution among pulmonologists across different diagnostic categories of PFT patterns. The green dot represents the mean value of the data, and the red line represents the median value. PFT, pulmonary function test

The box plot presents the distribution of prediction accuracy of different pulmonologists (Figure 3). It is clear that the accuracy of the pulmonologists varies greatly in making the right diagnosis. To protect the identities of the pulmonologists, no names have been disclosed.

PFT pattern interpretations

PFT pattern interpretation accuracy was made by eight senior pulmonologists against the gold pattern for 65.82%. The accuracy of individual pulmonologists varied from 54% to 79%.

Identification of categories

Pulmonologists struggled with diagnosing the categories moderate, moderately severe, severe, and others, with sensitivities of 47.73%, 44.01%, 60.34%, and 29.17%, respectively. The detailed confusion matrix is presented in Table 5.

**Table 5:** Confusion matrix of patterns interpretation: pulmonologists vs. gold pattern (N = 3,520). Data are presented as *n*. There are 2,317 (65.82%) correctly suggested diagnoses (true positive in bold). PPV, positive predictive value; NPV, negative predictive value.

			Gold pattern							Total
			Normal	Obstruction					Others	
				Mild	Moderate	Moderately severe	Severe	Very severe		
Pulmonologist pattern	Normal		225	40	55	15	5	0	1	341
	Obstruction	Mild	9	925	94	27	13	2	3	1,073
		Moderate	6	25	336	53	17	4	8	449
		Moderately severe	4	12	103	250	46	11	25	451
		Severe	2	8	67	103	280	27	50	537
		Very severe	1	4	31	75	69	238	66	484
	Others		1	2	18	45	34	22	63	185
Total			248	1016	704	568	464	304	216	3,520
Sensitivity (%)			90.73	91.04	47.73	44.01	60.34	78.29	29.17	-
Specificity (%)			96.45	94.09	95.99	93.19	91.59	92.35	96.31	-
PPV (%)			65.98	86.21	74.83	55.43	52.14	49.17	34.05	-
NPV (%)			99.28	96.28	88.02	89.64	93.83	97.83	95.41	-

Upon evaluation of the PFT pattern diagnosed by the AI algorithm against the gold pattern, an accuracy of 86.59% was achieved. AI results were interpreted using the most accurate algorithm, i.e., Decision Tree. AI algorithms also struggled with the interpretation of other categories. The detailed confusion matrix is presented in Table 6.

**Table 6:** Confusion matrix of patterns interpretation: AI vs. gold pattern Data are presented as *n*. There are 381 (86.59%) correctly suggested diagnoses (true positive in bold). PPV, positive predictive value; NPV, negative predictive value; AI, artificial intelligence

			Gold pattern							Total
			Normal	Obstruction					Others	
				Mild	Moderate	Moderately severe	Severe	Very severe		
AI interpreted pattern	Normal		28	5	1	0	0	0	0	34
	Obstruction	Mild	2	115	3	1	0	0	0	121
		Moderate	0	4	78	2	2	0	1	87
		Moderately severe	1	2	2	62	4	1	4	76
		Severe	0	1	3	2	48	1	2	57
		Very severe	0	0	1	2	3	35	5	46
	Others		0	0	0	2	1	1	15	19
Total			31	127	88	71	58	38	27	440
Sensitivity (%)			90.32	90.55	88.64	87.32	82.76	92.11	55.56	-
Specificity (%)			98.53	98.08	97.44	96.21	97.64	97.26	99.03	-
PPV (%)			82.35	95.04	89.66	81.58	84.21	76.09	78.95	-
NPV (%)			99.26	96.24	97.17	97.53	97.39	99.24	97.15	-

## Discussion

This study explored the possibility of leveraging the accuracy of the machine learning/AI algorithm to predict the PFT pattern using limited clinical data and spirometry. It also explored the consistency and accuracy of pulmonologists' predictions based on the same information.

Demographic and clinical distribution

More cases were reported among men (60%) than women, likely because, in our region, men are more commonly exposed to work-related smoking habits. Xu et al. reported a 1:1 male-to-female ratio [8], while Moreno Mendez et al. observed a 56:44 ratio [9].

In this study, 87% of participants reported changes in sputum, compared to 43.06% in the study by Xu et al. [8]. Cough was observed in 40% of cases in our study, whereas Xu et al. reported it in 78.21% of cases [8]. Dyspnea was reported in 88% of cases in this study, while Xiao et al. documented it in only 30.69% of cases [10].

Inter-rater variability

With a Fleiss's kappa of 0.46, indicating moderate agreement, it is evident that substantial disagreement exists among the patterns interpreted by different pulmonologists. This reinforces the notion that human interpretation is subject to variation due to many influencing factors.

This study identifies significant variability in the accuracy of diagnoses made by pulmonologists. Various factors may contribute to this variation, including personal experience, subjective assessment, and limited availability of clinical information. In their study, Topalovic et al. also observed a notable variation in diagnostic accuracy among pulmonologists [11].

Pattern interpretation accuracy

In this study, all 440 cases were interpreted both by eight pulmonologists and AI algorithms. The collective accuracy of pattern interpretations for all pulmonologists was 65.82%, and the accuracy of the AI algorithm was 86.59%. Pulmonologists could accurately interpret categories of normal, mild, and very severe obstruction. However, they struggled to interpret the moderate, moderately severe, and severe obstruction categories. This could be because it is easier to interpret ends of the spectrum. However, intermediate categories become more subjective and depend heavily on an individual’s experience and expertise.

In a study by Topalovic et al., 120 pulmonologists with varied work experience worked on their research [11]. The accuracy of pulmonologists was 74.4%, and that of the AI algorithm was 100%. However, their AI algorithm was trained not just on clinical and spirometry data but also included test data of body plethysmography and diffusion capacity. Access to more test data and parameters could be the reason for the higher accuracy of their pulmonologists and AI algorithms.

Das et al. conducted a study to assess the impact of using explainable AI algorithms over regular AI [12]. Using 24 cases, they estimated that accuracy increased from 62.5% to 87.5%. Bhattacharjee et al. used AI to classify obstructive and non-obstructive pulmonary diseases and achieved an accuracy of 83.7% [13].

Utility of AI

The utility of automatic assistance in healthcare is not a new concept. It is finding increasing space in many areas of healthcare, such as X-ray and mammography interpretation, automatic prescription suggestion, automatic ECG diagnosis, etc. Ribeiro et al. explored automatic diagnosis of the 12-lead ECG [14].

Advantages and limitations of AI

Currently, pulmonologists interpret the pattern based on spirometry results and clinical data. They tend to follow cutoffs for various parameters, such as FEV1. However, due to the availability of varied guidelines and myriad PFT test combinations, it becomes very challenging to achieve higher accuracy for them. 

AI algorithms, on the other hand, take all these data points and elevate them to a higher dimension of relationships. The more data points are used to train the AI algorithm, the more bias-free and robust it will be. Once such a trained model is ready, any new patient data will be elevated to a higher plane and be classified with very high accuracy [15]. AI algorithms offer quick, consistent, and repeatable diagnoses. Additionally, AI algorithm interpretations improve with time and experience [16].

However, the lack of clinical context and the unavailability of international guidelines limit the wider adoption of AI [17]. AI algorithms also face challenges related to data privacy and bias, and the need for human expertise must be addressed for the responsible and effective implementation of AI in healthcare [18].

Limitations

AI algorithms require large samples covering a myriad of spirometry and clinical feature combinations. For a few PFT patterns, more cases were needed to train and test AI algorithms. With more details, such as patient history, clinical examinations, radio imaging, etc., pulmonologists can increase their accuracy of pattern interpretation. In this study, the confidence level of the diagnosis made by pulmonologists was not captured. This could bring an additional dimension to the study.

## Conclusions

Based on this study, we can conclude that PFTs, when interpreted by pulmonologists with limited clinical and spirometry data, have lower accuracy and higher variability. AI algorithms can consistently produce high accuracy with the limited information in hand. This could be pivotal in the adoption of such technology among clinicians, especially in resource-constrained regions.

## Appendices

Appendix: Pulmonary Function Test - Week 10 - Quiz 2

DEMOGRAPHIC PARAMETERS: 

1. Gender:

2. Age:

3. BMI:

SMOKING CHARACTERISTICS:

1. Current Status: 

2. Length of Smoking: 

3. Intensity of Smoking:

SPUTUM:

1. Color: 

2. Viscosity: 

3. Consistency: 

*DYSPNEA (**Based on the mMRC scale)*:*

*COUGH* (*Based on the Cough Severity Index):

SPIROMETRIC PARAMETERS:

1. FEV1: 

2. FVC:

3. FEV1/FVC

Based on the above parameters, please suggest a diagnosis for the following:

1. PFT Pattern: Normal/Obstruction/Restriction/Mixed

2. PFT Pattern Severity (Only when PFT Pattern is Obstruction): Mild/Moderate/Moderately Severe/Severe/Very Severe 
